# Whole Genome Sequence–Based Surveillance of Human Clinical *Listeria monocytogenes* Isolates From Switzerland, 2019–2024

**DOI:** 10.1093/ofid/ofag222

**Published:** 2026-04-17

**Authors:** Magdalena Nüesch-Inderbinen, Jule Anna Horlbog, Nicole Cernela, Marc J A Stevens, Roger Stephan

**Affiliations:** Institute for Food Safety and Hygiene, Vetsuisse Faculty, University of Zurich, Zurich, Switzerland; National Centre for Enteropathogenic Bacteria and Listeria, Vetsuisse Faculty, University of Zurich, Zurich, Switzerland; Institute for Food Safety and Hygiene, Vetsuisse Faculty, University of Zurich, Zurich, Switzerland; National Centre for Enteropathogenic Bacteria and Listeria, Vetsuisse Faculty, University of Zurich, Zurich, Switzerland; Institute for Food Safety and Hygiene, Vetsuisse Faculty, University of Zurich, Zurich, Switzerland; Institute for Food Safety and Hygiene, Vetsuisse Faculty, University of Zurich, Zurich, Switzerland; Institute for Food Safety and Hygiene, Vetsuisse Faculty, University of Zurich, Zurich, Switzerland

**Keywords:** cluster types, core genome multilocus sequence typing, disease clusters, *Listeria monocytogenes*, listeriosis

## Abstract

**Background:**

*Listeria monocytogene*s is a foodborne pathogen that poses a significant public health risk due to the high case-fatality rate of listeriosis. Whole genome sequencing (WGS) is critical for identifying outbreak clusters and enables high-resolution characterization of isolates.

**Methods:**

We collected 335 *L monocytogenes* isolates from human cases of listeriosis occurring in Switzerland between 2019 and 2024. WGS was used to identify disease clusters and characterize the phylogenetic relatedness, virulence profiles, and antimicrobial resistance genes of the isolates.

**Results:**

The majority (77%) of the cases involved patients aged ≥65 years and was associated with bacteremia. The 5 major clonal complexes (CCs) were CC1, CC4, CC6, CC8, and CC388. The isolates belonged to 35 infection clusters, including 3 large outbreak clusters of 21 to 37 isolates per cluster. Nine smaller clusters included isolates highly related to food isolates obtained up to 4 years earlier. Multiple singly occurring isolates were linked to international outbreaks. WGS identified variations within key virulence factors including premature stop codons in InlA, length polymorphisms of ActA, and amino acid substitutions within PrfA. Hypervirulence associated with *Listeria* pathogenicity island (LIPI)-3 was found predominantly among CC1, CC4, and CC6 isolates. LIPI-4 was present in CC4 and CC388, and in infrequently occurring ST32, ST213, ST217, ST220, ST382, and ST1460.

**Conclusions:**

High-resolution typing of *L monocytogenes* is effective for the detection of disease clusters and for linking cases to international outbreaks. The identification of rare hypervirulent lineages demonstrates the importance of investigating the phylogenetic relatedness and virulence profiles of clinical *L monocytogenes*.

*Listeria monocytogenes* is an opportunistic and facultative intracellular foodborne pathogen that causes a variety of diseases that range from gastroenteritis to invasive listeriosis, with immunocompromised persons, older adults, and pregnant women considered to be vulnerable groups and at particular risk for infection [1]. The primary route of exposure of humans to *L monocytogenes* is the consumption of contaminated food, predominantly ready-to-eat food, fresh raw produce, and animal-derived food products that are consumed raw [2].

*Listeria monocytogenes* is categorized into 4 major evolutionary lineages (I to IV) and 14 lineage-related serotypes [3, 4]. While all *L monocytogenes* are considered potentially pathogenic, most (>95%) clinical isolates belong to serotypes 1/2b and 4b within lineage I and 1/2a within lineage II [3]. Multilocus sequence typing (MLST) based on the sequences of 7 housekeeping genes further subdivides the above categories into sequence types (STs), which group into distinct clonal complexes (CCs) that may consist of a single ST or multiple closely related STs [5]. Certain CCs, including hypervirulent isolates assigned to CC1, CC2, CC4, and CC6, are overrepresented among clinical isolates from invasive listeriosis cases [3, 6]. Currently, core genome–based MLST (cgMLST) is the most widely accepted approach for high-resolution typing of *L monocytogenes* and for the identification of clusters [6, 7].

A variety of virulence factors (VFs) enable *L monocytogenes* to invade and replicate within mammalian host cells, including key VFs such as InlA, which mediates bacterial adherence and the invasion of host cells; the pore-forming toxin listeriolysin O; phospholipases PlcA and PlcB, which promote vacuolar lysis; and ActA, which provides motility within the host cell cytosol and the cell-to-cell spread [1]. These and other VFs are located throughout the chromosome in operons and *Listeria* pathogenicity islands (LIPIs) and are coordinately expressed under the control of the PrfA transcriptional regulator which is located in LIPI-1 [1]. In addition, genotype-specific virulence traits have been described, the most important being LIPI-3, which comprises a gene cluster involved in the production of listeriolysin S, and LIPI-4, which contains 6 genes encoding cellobiose-type phosphotransferase systems that promote neural and placental listeriosis [6]. LIPI-3 is found predominantly among lineage I isolates, while LIPI-4 is associated with hypervirulence of CC4, CC87, and CC388 isolates and is involved in mediating neurovirulence and placental virulence [1, 8, 9]. Isolates carrying LIPI-4 are therefore of high clinical relevance. Other genes related to virulence are located within chromosomal *Listeria* genomic islands (LGI-1, LGI-2, and LGI-3), which may also contain resistance to heavy metals and increased tolerance to disinfectants [10].

Listeriosis is treated with ß-lactam antibiotics, usually in combination with an aminoglycoside, or alternatively, with co-trimoxazole (trimethoprim-sulfamethoxazole) [10]. Because the overall prevalence of acquired antimicrobial resistance (AMR) is low among clinical *L monocytogenes*, this reference treatment continues to be effective [10].

The incidence of invasive listeriosis is relatively low as reflected in the notified cases, which lie between 0.24 and 0.67 patients per 100 000 inhabitants in Europe and the United States [11, 12]. However, the case-fatality rate of approximately 20% is one of the highest for foodborne diseases [2]. The severity of *L monocytogenes* infection and the increasing number of foodborne outbreaks worldwide is a major public health burden, highlighting the need for increased understanding of the genomic diversity and virulence of this pathogen [13].

In Switzerland, listeriosis is a notifiable disease [11]. The Swiss National Reference Centre for Enteropathogenic Bacteria and *Listeria* (NENT) receives, collects, and routinely sequences clinical *L monocytogenes* isolates from diagnostic laboratories in Switzerland. Here, we present a comprehensive analysis of the genomic characteristics of clinical *L monocytogenes* from listeriosis patients in Switzerland between 2019 and 2024 and contextualize the genomes of the isolates with international and domestic outbreaks and analyze virulence variations of clones of international importance.

## MATERIALS AND METHODS

### Bacterial Isolates and Epidemiological Data

A total of 335 deduplicated (unique) *L monocytogenes* isolates from human cases of listeriosis in Switzerland were forwarded to the NENT for routine Illumina sequencing. Patient information including age, sex, and specimen source was collected from the submission forms as outlined in Supplementary Appendix 1. Information on hospitalization, underlying medical conditions, and infection outcome was not available for reasons of data protection.

Patients were classified according to their age to reflect different risk groups, which included infants (0 year of age), children and teenagers (1–19 years), adults (20–45 years), midlife adults (46–64 years), and older adults (≥65 years).

Cases were classified as (*i*) bacteremia, (*ii*) neurolisteriosis, (*iii*) pregnancy–associated and neonatal listeriosis, or (*iv*) focal forms of listeriosis, as detailed in Supplementary Appendix 1 and established by Koopmans et al [1]. In the absence of case histories, samples taken from pregnant women and of infants could not be matched and were counted as single cases.

### Whole Genome Sequencing

Genomic DNA was extracted using the DNeasy Blood and Tissue Kit (Qiagen). Sequencing libraries were prepared using the Illumina DNA Prep(M) Tagmentation kit (Illumina) and sequencing was performed on an Illumina MiSeq sequencer (Illumina). Reads were quality filtered using FastQC (https://www.bioinformatics.babraham.ac.uk/projects/fastqc/), and assemblies were generated using Skesa v2.5.1 [14], with a contig size cutoff >500 bp (Supplementary Appendix 1). Molecular serotypes and sequence types (STs) were determined in accordance with the BIGSdb–*L monocytogenes* platform (https://bigsdb.pasteur.fr/listeria). The cgMLST clusters and minimum spanning trees were calculated in SeqSphere [7]. A threshold of ≤10 allele differences was used for cluster definition, as recommended by Ruppitsch et al [7]. Isolates were compared based on cgMLST to food-associated isolates sequenced at the NENT during 2008–2024.

Virulence factors were identified using the Virulence Factor Database (VFDB) [15]. Premature stop codons (PMSCs) within the *inlA* genes, polymorphisms within the *actA* genes, and mutations in the *prfA* genes were identified using MAFFT v7.526 [16]. Mutations in the stress-inducible SigB operon was analyzed using parsnp with default settings [17]. Antimicrobial resistance genes and genes associated with biocide tolerance were detected using rgi v5.2.0 from the Comprehensive Antibiotic Resistance Database (CARD) [18], as detailed in Supplementary Appendix 1.

## RESULTS

### Demographic Features and Clinical Manifestations of *L monocytogenes* Infections

Between 2019 and 2024, a total of 335 of *L monocytogenes* isolates were received at the NENT, with each isolate allocated to a notified case. Of the 335 patients, 144 (43%) were female and 199 (57%) were male. The median age was 76 years (range, 0–95 years). A total of 8 (2%) were infants (0 years old), 24 (7%) were between 20 and 45 years old, and 44 (13%) belonged to the age group 46–64 years. A total of 259 cases (77%) occurred in patients ≥65 years of age (Table 1). There were no cases among children and teenagers (1–19 years).

**Table 1.** Key Characteristics of 335 Listeriosis Cases in Switzerland, 2019–2024

Culture Site	Clinical Form of Listeriosis	No. of Isolates (n = 335)	Sex		Age Group, y				
			Male (n = 193)	Female (n = 142)	0^a^ (n = 8)	1–19 (n = 0)	20–45 (n = 24)	46–64 (n = 44)	≥65 (n = 259)
Blood	Bacteremia	261	155	106	0	0	11	33	217
Cerebrospinal fluid	Neurolisteriosis	22	9	13	0	0	2	6	14
Amnion	Materno-fetal infection	1	0	1	0	0	1	0	0
Cervical swab	Materno-fetal infection	1	0	1	0	0	1	0	0
Placenta	Materno-fetal infection	5	0	5	0	0	5	0	0
Blood	Materno-fetal infection	6	2	4	6	0	0	0	0
Lung tissue	Materno-fetal infection	1	1	0	1	0	0	0	0
Stool/meconium	Materno-fetal infection	1	1	0	1	0	0	0	0
Other^b^	Focal infection^c^	16	11	5	0	0	3	4	9
Unknown	Unknown	17	13	4	0	0	1	0	16
Stool	Noninvasive infection	4	1	3	0	0	0	1	3

^a^Isolates from 0-year-old infants were considered to be from materno-fetal infections. They were counted as separate cases of listeriosis.

^b^Other culture sites included arterial heart biopsy, ascites, bile, pleural fluid, pus, skin lesion, synovial fluid, and urine.

^c^Focal infection comprised abscesses (n = 5), peritonitis (n = 3), joint infections (n = 2), pleural cavity infections (n = 2), biliary tract infections (n = 1), cardiovascular infections (n = 1), urinary tract infections (n = 1), and skin infections (n = 1).

Overall, 261 (78%) of the cases were associated with bacteremia, and 22 (7%) were cases of neurolisteriosis (Table 1). A total of 15 (4%) were pregnancy-related: 8 occurred in infants (0 years old) and 7 in women aged 20–45 years (Table 1). Focal infections were noted in 16 of 335 cases (5%) (Table 1). A total of 4 cases (1%) involved noninvasive listeriosis, and for 17 cases (5%), the site of isolation was unknown (Table 1 and Supplementary Appendix 2, Supplementary Table 1).

### Population Structure and Phylogenetic Relatedness of Clinical *L monocytogenes*

Molecular serotyping identified 160 (48%) isolates belonging to serotype 4b and 155 (46%) belonging to serotype 1/2a (Supplementary Appendix 2, Supplementary Table 1). A further 18 (5%) were of serotype 1/2b, whereas only 2 (1%) were serotype 1/2c (Supplementary Appendix 2, Supplementary Table 1). MLST revealed a total of 60 different STs that grouped into 40 CCs (Supplementary Appendix 2, Supplementary Table 1). The 5 most prevalent STs were ST6 (n = 42 [12.5%]), ST1 (n = 43 [12.8%]), ST3141 (n = 37 [11%]), ST388 (n = 28 [8.4%]), and ST4 (n = 19 [6%]). Other frequently observed STs included ST7 (n = 11 [3.3%]), ST8, ST29, and ST37 (n = 9 [2.7%] each), and ST16 (n = 8 [2.4%]). Three previously unknown STs were assigned by the Institut Pasteur database curators (ST3391, ST3392, and ST3393) [19].

The 5 major CC types were CC8 (n = 54 [16%]), CC1 (n = 48 [14%]), CC6 (n = 42 [13%]), CC388 (n = 28 [8%]), and CC4 (n = 21 [6%]).

**Figure 1.**
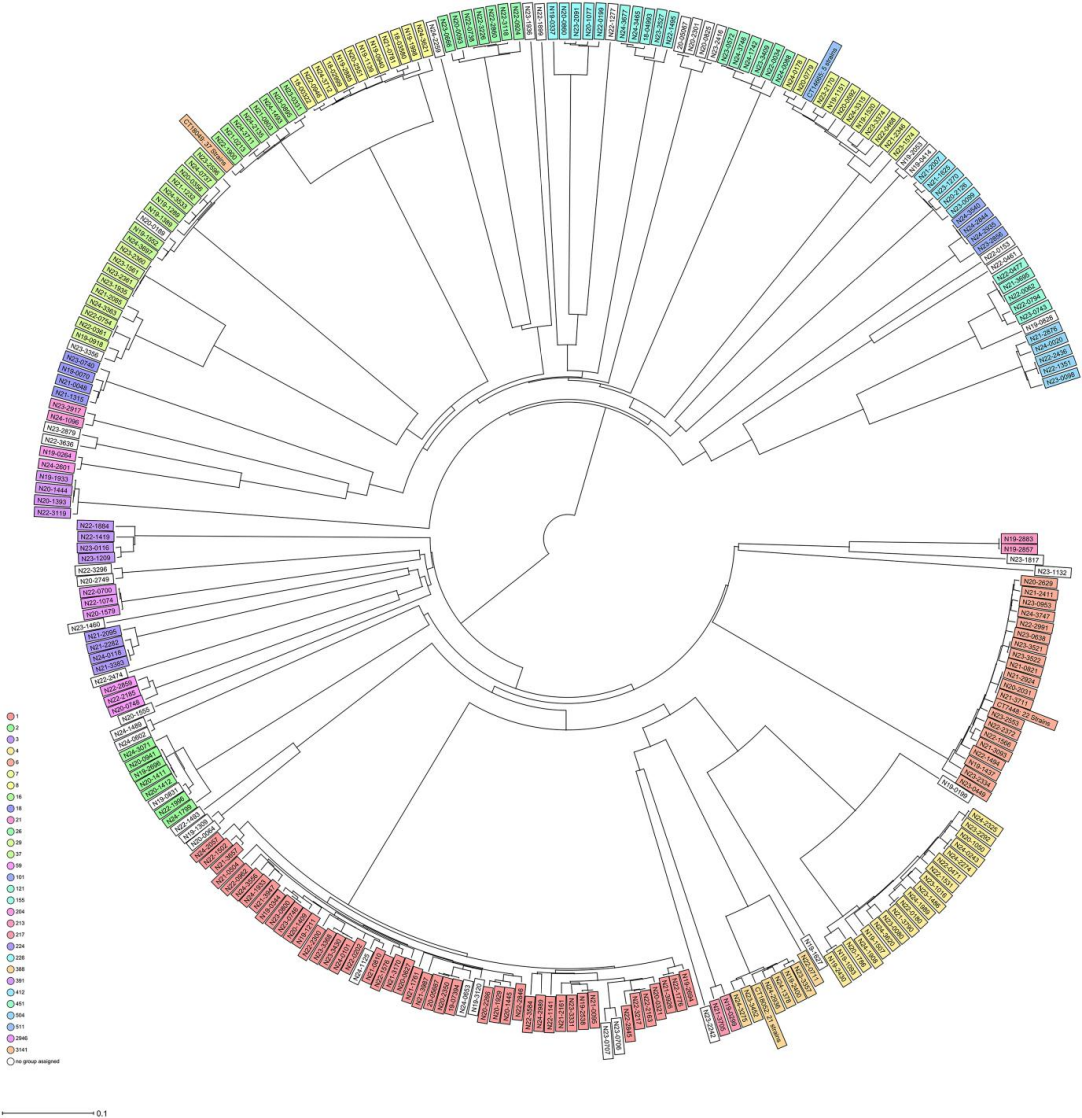
Phylogeny of 335 clinical *Listeria monocytogenes*. A core genome–based multilocus sequence typing matrix was converted to a neighborhood joining tree, which was visualized as midpoint-rooted tree in Figtree 1.4.4 (https://tree.bio.ed.ac.uk/software/figtree/). Colored boxes indicate sequence types. Isolates belonging to large outbreak clusters are indicated with number of isolates and the cluster number listed as in Table 2. The whole genome sequencing dataset and GenBank accession numbers for individual isolates are listed in Supplementary Appendix 2, Supplementary Table 5.

The 335 isolates belonged to 210 different cluster types (CTs) (Supplementary Appendix 2, Supplementary Table 1). Using a threshold of ≤10 allele differences revealed a total of 35 CT clusters consisting of 2 or more isolates (Figure 1 and Supplementary Appendix 2, Supplementary Table 1). The largest cluster (cluster 1 containing 37 isolates) belonged to ST3141 CT18049 (Table 2).

**Table 2.** Characteristics of Clinical *Listeria monocytogenes* Associated With Domestic Clusters and Cross-border Outbreaks, Switzerland, 2019–2024

MST Cluster^a^/Single Isolate	Cluster/Outbreak Designation	No. of Isolates	Year(s) of Isolation	Molecular Serotype	CC^b^	ST^c^	CT^d^	Food Source	Cross-border Outbreak	Reference
Cluster 1	LMCL-1	37	2022–2024	1/2a	CC8	ST3141	18049	Baker’s yeast	No	[20]
Cluster 2	LMCL-2	22	2020	4b	CC6	ST6	7448	Cheese products	No	[21]
Cluster 3	LMCL-3	21	2022	4b	CC388	ST388	18052	Smoked trout	No	[22]
Cluster 4	LMCL-4	5	2020–2021	1/2a	CC7	ST511	14665	Unknown	Unknown	This study
Cluster 5	LMCL-5	4	2022	1/2a	CC26	ST26	18051	Unknown	Unknown	This study
Cluster 6	LMCL-6	4	2019–2020	4b	CC2	ST2	7134	Unknown	Unknown	This study
Cluster 7	LMCL-7	4	2023	1/2a	CC29	ST29	19637	Soft cheese	No	This study
Cluster 8	LMCL-8	3	2024	1/2a	CC101	ST101	22236	Pork sausage	No	This study
Cluster 9	LMCL-9	3	2023–2024	4b	CC1	ST1	19660	Unknown	Unknown	This study
Cluster 10	LMCL-10	3	2020–2022	1/2b	ST2946	ST2946	14564	Unknown	Unknown	This study
Cluster 11	LMCL-11	3	2023–2024	1/2a	CC11	ST451	6376	Seafood/fish	No	This study
Cluster 13	LMCL-13	3	2021–2022	1/2a	CC121	ST121	16496	Meat	No	This study
Cluster 14	LMCL-14	3	2020–2021	4b	CC6	ST6	14566	Unknown	Unknown	This study
Cluster 15	LMCL-15	2	2021–2024	1/2a	CC8	ST16	15862	Unknown	Unknown	This study
Cluster 16	LMCL-16	2	2023	1/2a	CC8	ST16	19640	Unknown	Unknown	This study
Cluster 17	LMCL-17	2	2020–2023	1/2a	CC415	ST415	9312	Pork sausage	No	This study
Cluster 18	LMCL-18	2	2020–2023	1/2a	CC226	ST226	7614	Unknown	Unknown	This study
Cluster 19	LMCL-19	2	2022	4b	CC1	ST1	6522	Unknown	Unknown	This study
Cluster 20	LMCL-20	2	2019–2021	4b	CC1	ST1	10119	Pork sausage	No	This study
Cluster 21	LMCL-21	2	2024	4b	CC4	ST4	22226	Seafood/fish	No	This study
Cluster 22	LMCL-22	2	2019	4b	CC4	ST4	10115	Unknown	Unknown	This study
Cluster 23	LMCL-23	2	2021–2022	4b	CC4	ST4	16497	Unknown	Unknown	This study
Cluster 24	LMCL-24	2	2019–2023	4b	CC388	388	7607	Salad	No	This study
Cluster 25	LMCL-25	2	2021–2024	1/2b	CC224	224	15874	Unknown	Unknown	This study
Cluster 26	LMCL-26	2	2019	1/2a	CC8	ST8	10125	Unknown	Unknown	This study
Cluster 27	LMCL-27	2	2020–2021	1/2a	CC37	ST37	14558	Unknown	Unknown	This study
Cluster 28	LMCL-28	2	2022	1/2a	CC29	ST29	18046	Unknown	Unknown	This study
Cluster 29	Omikron1	2	2024	1/2a	CC155	ST155	1128	Salmon product	Yes	[23]
Cluster 30	2022-FWD-00102	2	2022	1/2a	CC475	ST504	16665^e^	Vegan cheese	Yes	[24]
Cluster 31	LMCL-31	2	2021–2022	4b	CC1	ST1	7612	Pasta	No	This study
Cluster 32	LMCL-32	2	2023	4b	CC1	ST1	19632	Unknown	Unknown	This study
Cluster 33	LMCL-33	2	2019	4b	ST213	ST213	4920	Unknown	Unknown	This study
Cluster 34	LMCL-34	2	2023	4b	CC6	ST6	19663	Unknown	Unknown	This study
Cluster 35	LMCL-35	2	2023–2024	4b	CC4	ST4	12418	Unknown	Unknown	This study
Single isolate	Beta2a	1	2021	1/2a	CC8	ST8	1247	Salmon product	Yes	[23]
Single isolate	Gamma6a	1	2019	1/2a	CC8	ST8	4172	Unknown	Yes	[25]
Single isolate	Kappa8	1	2020	4b	CC1	ST1	4961	Unknown	Yes	[25]
Single isolate	Ny9	1	2020	1/2a	CC415	ST394	13516	Smoked trout	Yes	[26]
Single isolate	Pi4	1	2022	1/2a	CC8	ST8	5004	Unknown	Yes	[25]

^a^MST cluster, minimum spanning tree–based clusters of clinical *L monocytogenes* containing ≤10 different alleles. Clusters were numbered in this study. Isolates occurring only once but representing part of cross-border outbreaks are referred to as single isolates.

^b^CC, clonal complex according to BIGSdb–*L monocytogenes* platform (https://bigsdb.pasteur.fr/listeria).

^c^ST, sequence type according to BIGSdb–*L monocytogenes* platform (https://bigsdb.pasteur.fr/listeria).

^d^CT, complex type according to Ruppitsch et al [7].

^e^CT16665 corresponds to CT11461 developed at the Institut Pasteur (https://bigsdb.pasteur.fr/) [10].

This cluster represents a large epidemiologically confirmed outbreak in Switzerland that occurred between 2022 and 2024 [20]. A Swiss commercial yeast factory and its production lines was the source of this outbreak, which was ended after the company initiated deep cleaning of all processing equipment [20].

Cluster 2 (22 isolates) consisted of ST6 CT7448 isolates that reflected a prolonged outbreak, which occurred during 2018–2020 and was traced back to the environmental contamination of a Swiss cheese dairy (Table 2) [21].

Cluster 3 (21 isolates) comprised ST388 CT18052 and represented a rapidly contained outbreak that was traced back to smoked trout from a local producer (Table 2) [22].

An additional 32 smaller clusters were identified, which consisted of between 2 and 5 isolates each (Table 2). Twenty-three isolates belonging to 9 clusters matched food isolates from the collection of sequenced *L monocytogenes* isolates within the database of the NENT (Table 2). Clusters 17 and 24 comprised isolates that were retrieved up to 4 years apart (Table 2).

A total of 9 isolates belonged to outbreaks reported in other countries: 2 isolates belonged to serotype 2a ST155 CT1128, which were part of the large multicountry Omikron1 outbreak linked to smoked salmon processed in Lithuania between 2016 and 2023 (cluster 29 in Table 2) [23].

A further, a singly occurring isolate typed 1/2a ST394 CT13516 matched the Ny9 outbreak involving cases from Germany, Austria, and Denmark between 2020 and 2021, caused by smoked rainbow trout from a Danish producer (Table 2) [26]. One isolate typed 1/2a ST8 CT1247 belonged to the prolonged Beta2a outbreak reported in Germany between 2016 and 2021 that was linked to salmon products (Table 2) [23]. Finally, 2 isolates of serotype 1/2a ST504 CT16665 were part of an outbreak associated with the consumption of vegan cheese, which affected patients in France, Belgium, Germany, and the Netherlands during 2022 (cluster 30 in Table 2) [24].

Three other isolates belonged to outbreaks Gamma6, Kappa8, and Pi4, which occurred in Germany between 2018 and 2021 but could not be traced back to their source (Table 2) [25]. For the remaining 19 clusters, no source was evident (Table 2).

### Virulence and AMR Profiles of Clinical *L monocytogenes* Isolates

All isolates harbored the invasion gene *inlA* (Supplementary Appendix 2, Supplementary Table 1). PMSCs within InlA were present in 11 isolates including all ST9 and ST121, and several other STs (Supplementary Appendix 2, Supplementary Table 3). Compared with the non-PMSC InlA, which contained 800 amino acids (AAs), the most observed truncated InlA consisted of 491 AAs, present in ST121 isolates (Supplementary Appendix 2, Supplementary Table 1).

All isolates possessed complete LIPI-1 comprising *actA*, *plcB*, *mpl*, *prfA*, *plcA*, and *hly* (V, Supplementary Table 3). Within LIPI-1, length polymorphisms in the *actA* gene were identified in 119 isolates (Supplementary Appendix 2, Supplementary Table 4). The phylogenetic tree showed a consistent pattern between isolates’ STs and their genetic polymorphisms (Supplementary Figure 1). In 3 isolates (N22-1419 [ST3], N24-1493 [ST16], and N24-3275 [ST388]), the *actA* gene could not be fully analyzed due to interruptions within the contigs (data not shown).

Amino acid substitutions in the PrfA protein were found at 5 positions: Y11H in 3 isolates belonging to CC415, L110I in 2 CC31 isolates, T165I in 1 CC26 isolate, S184T in 2 CC1 isolates, and K197N in 6 isolates (4 CC18 and 2 CC21) (Supplementary Appendix 2, Supplementary Table 1). No isolates had >1 mutation. Remarkably, isolate N23-1121, belonging to ST3141 and cluster 1, had a G95A mutation causing a premature stop and resulting in truncated, likely inactive PrfA of 31 AAs.

LIPI-3 was found in 129 isolates (Table 3). The proportion of isolates harboring LIPI-3 was highest among neuroinvasive and pregnancy-associated isolates (13/22 [59%] and 8/15 [53%]), and included all CC1, CC4, and CC6 isolates (Table 3 and Table 4). Incomplete LIPI-3 was characterized by the lack of *llsB* in 1 isolate (N22-1899 [ST768]) (Supplementary Appendix 2, Supplementary Table 3). Complete LIPI-4 was identified in 59 isolates, including 5 of 22 (23%) of the neuroinvasive isolates and 3 of 15 (20%) of the pregnancy-associated isolates (Table 3). LIPI-4 was present in isolates belonging to CC4, in all CC338 isolates (which included outbreak cluster 3), and in 9 further, infrequently occurring CCs and STs, some of which additionally co-harbored LIPI-3 (Table 4).

**Table 3.** Key Genetic Factors Associated With Hypervirulence Among 335 *Listeria monocytogenes* From Cases in Switzerland, 2019–2024

Culture Site	Clinical Form of Listeriosis	No. of Isolates (N = 335)	LIPI-3^c^, No. (%)	LIPI-4^c^, No. (%)
Blood	Bacteremia	261	94 (36)	44 (17)
Cerebrospinal fluid	Neurolisteriosis	22	13 (59)	5 (23)
Placenta and associated sites^a^	Materno-fetal infection	15	8 (53)	3 (20)
Other^b^	Focal infection	16	6 (38)	2 (13)
Unknown	Unknown	17	7 (41)	3 (18)
Stool	Noninvasive infection	4	1 (25)	2 (50)

^a^Associated sites included amnion, cervical swab, neonatal blood, neonatal lung tissue, and stool/meconium.

^b^Other culture sites included arterial heart biopsy, ascites, bile, pleural fluid, pus, skin lesion, synovial fluid, and urine.

^c^LIPI, *Listeria* pathogenicity island. Only isolates containing complete LIPIs are listed. LIPI-3 is composed of 8 genes (*llsAGHXBYDP*), and LIPI-4 contains LM9005581_70009, LM9005581_70010, LM9005581_70011, LM9005581_70012, LM9005581_70013, and LM9005581_70014 [27].

**Table 4.** Key Genetic Factors Associated With Hypervirulence Among *Listeria monocytogenes* From Cases in Switzerland, 2019–2024

Lineage	Serotype	Clonal Complex	Sequence Type	No. of Isolates (N = 335)	LIPI-3^a^, No. (%)	LIPI-4^a^, No. (%)
I	4b	CC1	ST1, ST278, ST1594, ST3372, ST3392	48	48 (100)	0
I	1/2b	CC3	ST3	4	4 (100)	0
I	4b	CC4	ST4, ST219, ST397	21	21 (100)	21 (100)
I	4b	CC6	ST6	42	42 (100)	0
I	4b	CC388	ST388	28	0	28 (100)
I	1/2b	CC18	ST498	1	1 (100)	0
I	4b	CC32	ST32	2	0	2 (100)
I	4b	CC54	ST54	1	1 (100)	0
I	4b	CC183	ST382	1	1 (100)	1 (100)
I	4b	CC217	ST217	2	2 (100)	2 (100)
I	4b	CC220	ST220	1	0	1 (100)
I	1/2b	CC224	ST224	4	4 (100)	0
I	4b	CC389	ST389	2	2 (100)	0
I	4b	CC666	ST666	1	1 (100)	0
I	4b	None	ST213	2	2 (100)	2 (100)
I	1/2b	CC517	ST517	1	0	1 (100)
I	1/2b	None	ST1460	1	0	1 (100)

^a^LIPI, *Listeria* pathogenicity island. Only isolates containing complete LIPIs are listed. LIPI-3 is composed of 8 genes (*llsAGHXBYDP*), and LIPI-4 contains LM9005581_70009, LM9005581_70010, LM9005581_70011, LM9005581_70012, LM9005581_70013, and LM9005581_70014 [27].

Intrinsic AMR genes *lin* (encoding resistance to lincosamides) and *mprf* (resistance to cationic peptides and defensins) were present in all isolates, whereas the fosfomycin resistance gene *fosX* was lacking in 3 CC1 isolates belonging to cluster 9, and the quinolone resistance efflux pump gene *norB* was missing among 4 C155 isolates, 2 of which were part of the Omikron1 outbreak (Supplementary Appendix 2, Supplementary Table 5). No known mutations in core genes associated with AMR to ciprofloxacin (*gyrAB*/*parC*), rifampicin (*rpoD*), or streptomycin (*rrn*) were detected. Notably, no acquired AMR genes were found.

Genes conferring increased tolerance to quaternary ammonium compounds that are used as disinfectants in the food industry included *qac* in 12 isolates mainly belonging to CC121, and the *brcABC* operon in 6 isolates (Table 5). Complete LGI-2 containing the full set of 34 loci was detected in 13 isolates belonging to CC2, CC4, CC14, and CC204, while a complete LGI-3 comprising 29 loci was found in only 1 CC101 isolate (Table 5). Multiple isolates contained incomplete LGI-2 or LGI-3, whereas LGI-1 was not detected (Supplementary Appendix 2, Supplementary Table 5). We found 2 nonsynonymous mutations in SigB compared to reference strain EgdE (ATCC BAA-679): V107M in 1 isolate, and Y216F in 182 isolates (notably all serotype IV) (Supplementary Table 2). Mutations in *sigB* that enhance stress tolerance have been described in food and clinical isolates [28]. Whether the mutations impact stress tolerance is not known.

**Table 5.** Key Genetic Factors Associated With Acquired Virulence Traits and Genes Associated With Increased Tolerance to Biocides Among *Listeria monocytogenes* From Cases in Switzerland, 2019–2024

Lineage	Serotype	Clonal Complex	Sequence Type	No. of Isolates (N = 335)	No. (%) of Isolates Harboring^a^			
					LGI-2^b^	LGI-3^b^	*qac*	*bcrABC*
I	4b	CC1	ST1	43	0	0	1 (2)	0
I	4b	CC2	ST2, ST1531	8	8 (100)	0	3 (38)	0
I	4b	CC4	ST4	19	2 (11)	0	0	0
I	4b	CC6	ST6	42	0	0	0	0
II	1/2a	CC8	ST8	9	0	0	2 (22)	0
II	1/2a	CC14	ST14	1	1 (100)	0	0	0
II	1/2a	CC31	ST325	2	0	0	0	1 (50)
II	1/2a	CC204	ST204	2	2 (100)	0	0	0
II	1/2a	CC101	ST101	4	0	1 (25)	0	0
II	1/2a	CC121	ST121, ST3391	6	0	0	6 (100)	0
II	1/2a	CC155	ST155	4	0	0	0	1 (25)
II	1/2a	CC321	ST321	1	0	0	0	1 (100)
I	1/2b	None	ST2946	3	0	0	0	3 (100)

^a^Only isolates containing complete *Listeria* genomic islands (LGIs) are listed.

^b^LGI-2 contains 34 loci, and LGI-3 contains 29 loci [10].

## DISCUSSION

Data on the population structure and virulence traits of *L monocytogenes* are crucial for mitigating the public health burden of listeriosis. We used whole genome sequencing (WGS)–based analysis to characterize 335 clinical *L monocytogenes* collected over 6 years in Switzerland. During the study period, most cases of invasive listeriosis manifested as bacteremia and affected patients ≥65 years of age, which corresponds to earlier data [1]. With almost 20% of the population aged ≥65 years and a rising life expectancy, Switzerland is an aging population [29], and listeriosis cases may become more common. There were no notifications of listeriosis among children or persons <20 years of age during the study period, which is consistent with prior studies, although listeriosis outbreaks have in rare occasions included schoolchildren [1].

*Listeria monocytogenes* CC1, CC4, CC6, CC8, and CC388 were prevalent. While CC1, CC4, and CC6 are international clones globally associated with human listeriosis [1, 6], the overrepresentation of CC8 (containing ST3141 CT18049) and CC388 (comprising ST388 CT18050) was country-specific and due to large outbreaks that occurred during this time [20, 22]. While *L monocytogenes* ST3141 is unique to Switzerland so far and therefore limited in its geographical distribution, ST388 CT8466 was reported in connection with a large outbreak in Spain in 2019 [30]. Although the Swiss and the Spanish ST388 were not closely related, these outbreaks highlight CC388 as a clone with the potential to become more widespread.

In addition to the large outbreak clusters, multiple listeriosis cases were represented by a total of 32 small clusters. Notably, 9 clusters with 2–5 cases each matched by cgMLST to a food isolate, in some cases, years apart. These observations suggest local or regional common sources and multiyear persistent contamination in food-associated environments such as farms, food processing plants, or retail establishments [31]. Small clusters with temporal scattering of clinical isolates have been reported from other countries, including France [27], Germany [25], Italy [32], the Netherlands [33], and the United States [34]. Therefore, identifying persisting isolates within regional food-associated environments can be key for source attribution and the protection of public health.

Further, multiple singly occurring cases were linked to international outbreaks that occurred in several European countries [23–26]. These findings indicate that single cases interpreted as sporadic cases (ie, cases that do not cluster with any other) tend not to be investigated as part of an outbreak, highlighting the need for data comparison and immediate information sharing at the European and international levels.

The results from our study expand previous findings on the occurrence of VFs and hypervirulent CCs among clinical *L monocytogenes*. All isolates contained key VFs. Truncated InlA, polymorphisms in ActA, and AA substitutions within PrfA were identified in multiple isolates, irrespective of their associated clinical manifestation. For example, PMSCs in InlA were found predominantly among ST9 and ST121, which are STs associated with low invasive activity but strong adaptability to survival in food and food production environments [1]. While the potential evolutionary benefits of loss of a full-length InlA is not fully understood [5], our data indicate that the absence of a full-length InlA does not exclude the potential to cause invasive listeriosis. Similarly, ActA polymorphisms correlated with the STs of the isolates. The distribution of these polymorphisms into distinct STs suggests a common ancestor for each of these groups of isolates, or ecological adaptations beyond their role of human pathogens. The high diversity of ActA in clinical isolates strongly suggests that polymorphisms do not substantially influence the virulence potential of *L monocytogenes*, supporting previous studies [35]. Last, alterations in PrfA were identified, but their effect is likely minimal. The activation of PrfA requires it to bind with the cofactor glutathione; however, none of the AA substitutions were in the regions that bind glutathione, and to the best of our knowledge, also not close to AAs that are known to affect PrfA activity [36].

Additional genomic traits associated with *L monocytogenes* virulence such as the LIPI-3 and LIPI-4 islands were present in multiple isolates, including CC1, CC4, and CC6, all of which are considered to be hypervirulent clones [6]. LIPI-4 is associated with CC4, which is more likely than other clones to cause infections of the central nervous system and placenta [6]. However, in this study, only a small proportion of the isolates associated with neurolisteriosis and materno-fetal listeriosis harbored LIPI-4, suggesting that other, host-associated factors may play a role in these disease manifestations.

Originally reported only in isolates from CC4 [6], the presence of LIPI-4 is increasingly reported in other CCs and STs worldwide [8]. For example, *L monocytogenes* CC183 ST382 co-harboring LIPI-3/LIPI-4, represented by 1 isolate in our study, is an emerging hypervirulent clone in North America that has been implicated in 3 produce-related outbreaks since 2013 [37]. Likewise, *L monocytogenes* CC217 ST217 from 2 pregnancy-associated isolates in this study was identified as a long-term persisting cluster of invasive *L monocytogenes* in New York State [34]. Further, in our study we found LIPI-3/LIPI-4 in a cluster of 2 pregnancy-associated *L monocytogenes* ST213, which was reported in 2 clinical cases in New York State in 2005 and 2019 [34]. Finally, we detected LIPI-4 in less common lineages such as ST32, ST220, and ST1460. Since there is a lack of information whether these minor STs normally carry LIPI-4, the identification in our study of rare lineages of hypervirulent *L monocytogenes* contributes to the understanding of the genetic diversity of isolates causing invasive listeriosis in humans.

Notably, the absence of transmissible AMR genes in the study isolates indicates that resistance to the main antimicrobials used in the treatment of listeriosis, such as ampicillin and gentamicin, is very low among clinical *L monocytogenes* in Switzerland. Our findings are in line with recent studies from France, Germany, Lithuania, and the United States, which showed low levels of acquired resistance to clinically important antimicrobials such as aminoglycosides, tetracycline, and erythromycin [10, 25, 34]. Our data provide further evidence for the efficacy of the current reference treatment of listeriosis. However, given the recent increase in multidrug-resistant (MDR) *L monocytogenes* in China, including the emergence of MDR LIPI-4 harboring hypervirulent clone CC87 in food [38], there remains the need to address the potential spread of AMR *L monocytogenes* across countries and continents.

This study has some limitations. First, due to data protection regulations, data on disease progression, clinical outcomes, and comorbidities were not available, limiting the clinical interpretation of our findings. Second, in this study we used the cgMLST scheme developed by Ruppitsch et al to determine CTs and perform cluster analysis [7]. For the comparison of isolates with internationally occurring CTs, it is important to keep in mind that the Pasteur scheme by Moura et al is another commonly used cgMLST scheme with a different CT nomenclature [19]. Thus, the detection of some clonally related isolates from locations abroad may have been missed due to different CT denominations. Despite this limitation, the data from this study enabled us to contextualize Swiss clinical *L monocytogenes* isolates with cross-border outbreaks and internationally occurring clones and lineages.

## CONCLUSIONS

Listeriosis primarily affects the older population. Guidance provided by health and food safety authorities should be followed at all levels of the food supply chain and within the healthcare setting to mitigate the risk of infection.

WGS-based high-resolution cluster typing of *L monocytogenes* has proven effective for outbreak and source detection in Switzerland, as well as for linking sporadic cases of listeriosis to outbreaks of occurring abroad. Our study emphasizes the importance of implementing WGS for routine *L monocytogenes* surveillance, which would ideally include harmonized cgMLST-based cluster typing schemes to facilitate communication between national and local authorities. Our data provide a comprehensive insight on the phylogenetic diversity and the virulence variants of human clinical *L monocytogenes*, including hypervirulent lineages outside of otherwise highly represented CCs.

## Supplementary Material

ofag222_Supplementary_Data

## Supplementary Data

Supplementary materials are available at *Open Forum Infectious Diseases* online. Consisting of data provided by the authors to benefit the reader, the posted materials are not copyedited and are the sole responsibility of the authors, so questions or comments should be addressed to the corresponding author.
