# Supplementary material for: Whole Genome Sequence–Based Surveillance of Human Clinical Listeria monocytogenes Isolates From Switzerland, 2019–2024
Source: Open Forum Infect Dis. 2026 Apr 17;13(5):ofag222. doi: 10.1093/ofid/ofag222 (PMC13126514; doi:10.1093/ofid/ofag222)
Supplement: ofag222_Supplementary_Data [file ofag222_supplementary_data.zip › R1.0 Supplementary Appendix 1 OFID.docx]

**Supplementary materials**

**Whole-genome sequence-based surveillance of human clinical *Listeria monocytogenes* isolates from Switzerland, 2019–20244**

Magdalena Nüesch-Inderbinen^1,2^, Jule Anna Horlbog^1,2^, Nicole Cernela^1^, Marc J.A. Stevens^1^, Roger Stephan^1*^

^1^ Institute for Food Safety and Hygiene, Vetsuisse Faculty, University of Zurich, Zurich, Switzerland

^2^ National Centre for Enteropathogenic Bacteria and *Listeria* (NENT), Vetsuisse Faculty, University of Zurich Zurich, Switzerland

*Author for correspondence: Prof Roger Stephan: [stephanr@fsafety.uzh.ch](mailto:stephanr@fsafety.uzh.ch), Winterthurerstrasse 272, CH-8057 Zürich, Switzerland.

Phone: +41 44 635 86 51

**Contents**

1. Supplementary Methods 4

2. Supplementary Figure 6

3. Supplementary References 8

# **1. Supplementary Methods**

**Classification of listeriosis cases**

Patient information including sex and age was collected from the submission forms of the forwarding laboratories. Data included specimen source as indicated on the submission forms (blood, cerebrospinal fluid, placenta, cervical or amnion specimens, and stool samples). Other sources included biopsy samples, ascites, bile, pleural fluid, pus, skin lesion, synovial fluid, and urine.

Bacteremia was defined when *L. monocytogenes* was isolated from blood, and neurolisteriosis was defined when *L. monocytogenes* was isolated from the cerebrospinal fluid (CSF). Pregnancy-associated and neonatal listeriosis cases were defined based on the isolation of *L. monocytogenes* from a clinical sample of a pregnant woman (cervical swab, amnion, or placenta), or an infant (0 year of age), respectively. Samples of pregnant women and of infants could not be matched and were counted as single cases. Other forms of invasive listeriosis were represented by *L. monocytogenes* isolated from less common sites such as joint tissue, peritoneal, lung, and pleural cavities, urinary and biliary tracts etc. (appendix 2, Table S1). Non-invasive listeriosis was defined based on the isolation of *L. monocytogenes* isolated from stool (excluding infants of 0 years of age). Duplicate isolates from the same patient were excluded.

**Whole genome sequencing and bioinformatic analysis**

*L. monocytogenes* isolates were grown overnight at 37°C on Columbia sheep blood agar (Difco Laboratories). Genomic DNA was isolated using the DNeasy Blood and Tissue Kit (Qiagen, Hilden, Germany). Sequencing libraries were prepared using the Illumina DNA Prep(M) Tagmentation kit (Illumina, San Diego, CA, USA) and sequencing was performed on an Illumina MiSeq sequencer (Illumina, San Diego, CA, USA) with 2 × 150 bp paired-end chemistry to a minimum coverage of 50x. Following a quality assessment with FastQC (https://www.bioinformatics.babraham.ac.uk/projects/fastqc/), assemblies were generated using Skesa v2.5.1 [1] in Shovill 1. 1.0 (github.com/tseemann/shovill) with default settings and a contig size cut off > 500 bp

(github.com/tseemann/shovill) and integrated into the Ridom SeqSphere + software version 5.1.0 (Ridom, Münster, Germany) [2]. Molecular serotypes, seven locus multi-locus sequence typing (MLST) sequence types (STs) were determined in accordance with the *L. monocytogenes* BIGSdb-*L. monocytogenes* platform (https://bigsdb.pasteur.fr/listeria).

**Core genome MLST and phylogenetics**

Core genome MLST analysis was done in the Ridom SeqSphere + software version 10.0.6 (Ridom, Münster, Germany) using the core genome defined by Ruppitsch et al. [2]. Molecular serotypes, seven locus multi-locus sequence typing (MLST) sequence types (STs) were determined in accordance with the *L. monocytogenes* BIGSdb-L. monocytogenes platform (<https://bigsdb.pasteur.fr/listeria>).

Phylogenetic trees were based on allele distance matrices calculated in SeqSphere the with “pairwise ignore missing values” mode for visualization of strain relatedness. Trees were plotted as midpoint rooted neighborhood joining tree in Figtree v1.4.4 (<https://github.com/rambaut/figtree>). Minimum spanning trees (MSTs) were generated in Ridom SeqSphere + version 10.0.6 and genetic clusters were defined as isolates containing ≤ 10 different alleles between a pair of isolates.

To place the study isolates into a wider context, *L. monocytogenes* sequences belonging to food products and food associated environments collected at the NENT during 2008–2024 were retrieved from the in-house database and compared based on cgMLST. In addition, sequences associated with outbreak strains from other countries available from public sequence databases were included for cgMLST comparison.

All databases and sequence data were downloaded from the corresponding servers in June 2025.

**Virulence factors and antimicrobial resistance genes**

Virulence factors (VFs) were identified by comparing the strain's predicted proteomes with the representative protein dataset from the Virulence Factor Database (VFDB) (<http://www.mgc.ac.cn/VFs/>) [3], in combination with the BIGSdb-Lm server from the Institut Pasteur [4]. DBs were downloaded in June 2025. A bi-directional best hit search was performed with the proteome and the respective database, using diamond v2.1.11.165 wit default setting [5] to identify VFs and applying a cut off of > 70% identity. The presence of Listeria pathogenicity islands was determined similarly, by comparing the proteome to the Listeria Genomic Islands DB from the Pasteur institute. The identification of premature stop codons (PMSCs) within the *inlA* genes, polymorphisms within the *actA* genes, and mutations in the *prfA* genes were identified by exporting the corresponding nucleotide sequences from SeqSphere + and aligning these sequences using MAFFT v7.526 (https://mafft.cbrc.jp/alignment/server/index.html) followed by manual inspection. Antimicrobial resistance genes were detected using the resistance gene identifier rgi v5.2.0 from the CARD Database with default settings ([http://arpcard.mcmaster.ca](http://arpcard.mcmaster.ca/)) [6], and a cut-off of at least 70% identity over 90% of the query length.

# **2. Supplementary Figure**

**Figure S1. Length polymorphisms in the *actA* gene of 119 clinical *L. monocytogenes***

A protein sequence alignment was made using mafft v7.526 and subsequently a tree was calculated using IQtree v2.0⁠⁠, with the option "-m MFP" for “extended model selection followed by tree inference” activated. A midpoint -rooted tree was visualized using Figtree v1.4.4⁠ (https://tree.bio.ed.ac.uk/software/figtree/). In three isolates (N22-1419 (ST3), N24-1493 (ST16) und N24-3275 ST388)), the *actA* gene could not be fully analyzed due to interruptions within the contigs (data not shown).

# **3. Supplementary References**

1. Souvorov A, Agarwala R, Lipman DJ. SKESA: strategic k-mer extension for scrupulous assemblies. Genome Biol 2018;**19:**153. https://doi.org/10.1186/s13059-018-1540-z.

2. Ruppitsch W, Pietzka A, Prior K et al. Defining and evaluating a core genome multilocus sequence typing scheme for whole-genome sequence-based typing of *Listeria monocytogenes*. J Clin Microbiol 2015;**53:**2869–76. https://doi.org/10.1128/JCM.01193-15.

3. Liu B, Zheng D, Zhou S, Chen L, Yang J. VFDB 2022: a general classification scheme for bacterial virulence factors. Nucleic Acids Res 2022;**50:**D912-17. https://doi.org/10.1093/nar/gkab1107.

4. Moura A, Criscuolo A, Pouseele H et al. Whole genome-based population biology and epidemiological surveillance of *Listeria monocytogenes*. Nat Microbiol 2016;**2:**16185. https://doi.org/10.1038/nmicrobiol.2016.185.

5. Buchfink B, Reuter K, Drost HG. Sensitive protein alignments at tree-of-life scale using DIAMOND. Nat Methods 2021; **18:**366-68. https://doi.org/10.1038/s41592-021-01101-x.

6. Alcock BP, Raphenya AR, Lau TTY et al. CARD 2020: antibiotic resistome surveillance with the comprehensive antibiotic resistance database. Nucleic Acids Res 2020;**48:**D517-25. https://doi.org/10.1093/nar/gkz935.
